# The Role of Gut Microbiota in the Efficacy and Side Effect Profile of Biologic Therapies for Autoimmune Diseases

**DOI:** 10.7759/cureus.71111

**Published:** 2024-10-08

**Authors:** Naeem Qusty, Anas Sarhan, Medhat Taha, Ahmed Alshanqiti, Albaraa Mohammed Almuteb, Aisha Tareq Alfaraidi, Hussein Ali Alkhairi, Manar Mohammed Alzahrani, Aishah Hanash A Alamry, Talal Qalil Bakheet Alomry, Omar Abdu Bannan, Mohammed Saeed Almaashi

**Affiliations:** 1 Department of Clinical Laboratory Sciences, Umm Al-Qura University, Makkah, SAU; 2 Department of Internal Medicine, Umm Al-Qura University, Makkah, SAU; 3 Department of Anatomy, Umm Al-Qura University, Al-Qunfudhah, SAU; 4 College of Medicine, Umm Al-Qura University, Al-Qunfudhah, SAU; 5 College of Medicine, Imam Mohammad Ibn Saud Islamic University, Riyadh, SAU; 6 College of Medicine, King Abdulaziz University, Jeddah, SAU; 7 College of Medicine, Taif University, Taif, SAU; 8 College of Medicine, Petre Shotadze Tbilisi Medical Academy, Tbilisi, GEO

**Keywords:** autoimmune diseases, biologic therapies, gut microbiota, microbial profiles, patient

## Abstract

The role of gut microbiota in influencing the efficacy and side effect profile of biological therapies for autoimmune diseases has gained increasing attention. Understanding these interactions is crucial for optimizing treatment outcomes and minimizing adverse events associated with biological therapies. This systematic review was conducted following the Preferred Reporting Items for Systematic Reviews and Meta-Analyses (PRISMA) guidelines. We comprehensively analyzed studies involving human subjects with autoimmune diseases treated with biological therapies. Data on gut microbiota composition, therapeutic efficacy, and side effect profiles were extracted and synthesized to assess the impact of microbiota on treatment outcomes.

Our review identified a significant relationship between gut microbiota composition and the efficacy of biological therapies. Specific bacterial taxa, such as Clostridiales and *Roseburia inulinivorans*, were associated with improved therapeutic responses, while alterations in microbiota were linked to increased adverse events. The predictive potential was demonstrated with microbiota signatures correlating with treatment success and side effects, highlighting the relevance of microbial profiles in therapeutic outcomes. The findings suggest that gut microbiota plays a pivotal role in modulating the efficacy and side effect profile of biological therapies for autoimmune diseases. Integrating microbiota assessments into clinical practice could enhance personalized treatment strategies and improve patient outcomes.

## Introduction and background

Autoimmune diseases, such as inflammatory bowel disease (IBD), which includes Crohn's disease and ulcerative colitis, are largely driven by an abnormal immune response to gut microbes in genetically susceptible individuals [1]. Risk factors such as diet, antibiotic use, and infections also contribute to disease onset and exacerbation [1]. Dysbiosis, or microbial imbalance, is a hallmark of IBD, with reduced levels of beneficial bacteria like *Firmicutes *and *Bacteroides *leading to compromised gut barrier function and heightened immune activation [2]. This chronic inflammation underscores the importance of gut microbiota in IBD pathology and therapeutic outcomes. Psoriasis, a chronic immune-mediated skin condition, is triggered by both genetic and environmental factors, including stress, infections, and lifestyle choices [3]. The gut-skin axis is increasingly recognized as influential in psoriasis, where gut dysbiosis contributes to systemic inflammation through microbial metabolites such as short-chain fatty acids (SCFAs) [4]. These metabolites modulate immune responses, potentially exacerbating skin inflammation. This emerging understanding of the gut’s role in psoriasis offers potential avenues for microbiota-targeted therapies to improve biologic treatment outcomes. Rheumatoid arthritis (RA), an autoimmune disorder characterized by joint inflammation, is influenced by genetic factors like the HLA-DRB1 gene as well as lifestyle factors such as smoking [5]. The gut-joint axis is a significant player in RA development, with research showing that reduced levels of beneficial bacteria like *Prevotella *increase gut permeability, allowing bacterial antigens to enter the bloodstream and provoke immune responses that trigger joint inflammation [5]. This connection highlights the role of gut microbiota in the immune dysregulation seen in RA, opening doors to personalized treatments targeting microbial imbalances. Recent guidelines for the management of IBD, psoriasis, and RA emphasize the growing importance of personalized medicine, particularly in relation to biological therapies. In IBD, updated guidelines from the American College of Gastroenterology (ACG) highlight the role of biologics such as TNF inhibitors, vedolizumab, and ustekinumab in achieving and maintaining remission, with recommendations for regular monitoring of response and adverse effects [1]. Psoriasis management guidelines from the American Academy of Dermatology (AAD) stress the need for biologic treatments, including IL-17, IL-23, and TNF inhibitors, tailored to disease severity and patient comorbidities, while also addressing the potential side effects like increased risk of infection [3]. Similarly, the American College of Rheumatology (ACR) guidelines for RA recommend early and aggressive treatment with biologics such as TNF inhibitors, IL-6 inhibitors, and JAK inhibitors, particularly for patients with high disease activity, while emphasizing the need to weigh the benefits against risks like serious infections and malignancies [5]. These guidelines across the three disorders reflect an increased focus on optimizing biological use to improve patient outcomes. However, response to these therapies varies significantly among patients, with some experiencing robust clinical improvement while others show partial or no response [6,7]. Additionally, the side effect profiles of biologics can be concerning, ranging from mild adverse reactions to serious infections and malignancies [8-11].

Recent research has increasingly highlighted the role of the gut microbiota - the diverse community of microorganisms residing in the gastrointestinal tract - as a crucial factor in modulating both the efficacy and safety of biologic therapies [12,13]. The gut microbiome is known to interact with the host immune system, influencing immune tolerance, inflammatory responses, and the overall balance of immune regulation [14-16]. Dysbiosis, or an imbalance in the gut microbial composition, has been implicated in the pathogenesis of various autoimmune diseases, suggesting that it may also affect treatment outcomes [17,18].

Several studies have explored the gut microbiota's potential role in shaping individual responses to biological therapies [19-21]. For instance, certain microbial profiles have been linked to enhanced therapeutic responses, while others are associated with increased susceptibility to side effects such as infections [22]. The bidirectional interaction between biologic agents and the microbiota suggests that microbiome-targeted interventions, such as probiotics, prebiotics, or fecal microbiota transplantation, may serve as adjunctive strategies to optimize therapeutic outcomes [22].

This systematic review aims to comprehensively analyze the existing evidence on the role of gut microbiota in the efficacy and side effect profile of biologic therapies for autoimmune diseases. By synthesizing current research, we seek to provide insights into how modulating the gut microbiome may improve patient outcomes and reduce adverse events associated with biologics. Understanding these interactions could pave the way for more personalized therapeutic approaches in the management of autoimmune conditions.

## Review

Methodology

This systematic review was conducted following the Preferred Reporting Items for Systematic Reviews and Meta-Analyses (PRISMA) guidelines. A comprehensive search of relevant studies was performed across multiple databases, including PubMed, Scopus, and Web of Science, to identify peer-reviewed articles published up to 2023 that investigated the role of gut microbiota in the efficacy and side effect profile of biologic therapies for autoimmune diseases. Keywords such as “gut microbiota,” “biologic therapies,” “autoimmune diseases,” “efficacy,” and “side effects” were used, along with appropriate Boolean operators to ensure a thorough search (Table 1). Articles were included if they met the following criteria: studies involving human subjects with autoimmune diseases treated with biological therapies, those reporting outcomes related to treatment efficacy and side effects, and studies assessing the gut microbiota's role in these outcomes. Studies that did not provide primary data, reviews, case reports, or those not available in English were excluded.

**Table 1:** Search strategies used for the studies

Search strategy	Database/tool	Search terms and keywords	Date range	Filters applied
1. Initial search	PubMed	"gut microbiota" AND "biologic therapies" AND "autoimmune diseases"	January 2010-August 2023	Human studies, English language
2. Broad search	Embase	"gut microbiome" AND "biologics" AND "autoimmunity"	January 2010-August 2023	Human studies, English language
3. Targeted search	Cochrane Library	"microbiota" AND "biologic therapy" AND "autoimmune conditions"	January 2010-August 2023	Systematic reviews, clinical trials
4. Expanded search	Scopus	"microbiome" AND "biologics" AND "autoimmune diseases"	January 2010- August 2023	Human studies, English language
5. Supplementary search	Google Scholar	"gut microbiota" AND "biologic agents" AND "autoimmune disorders"	January 2010-August 2023	No specific filters applied

Two independent reviewers screened titles and abstracts for eligibility, followed by a full-text review of selected studies. Any discrepancies between reviewers were resolved through discussion or by a third reviewer. Data extraction focused on study design, patient characteristics, type of biologic therapy used, methods of microbiota analysis, and outcomes related to efficacy and side effects. The risk of bias in the included studies was assessed using the Cochrane Risk of Bias tool for randomized controlled trials and the Newcastle-Ottawa Scale for observational studies. Data were synthesized qualitatively, and a narrative approach was used to present the findings, given the heterogeneity in study designs and outcome measures.

Results

General Characteristic

**Figure 1:**
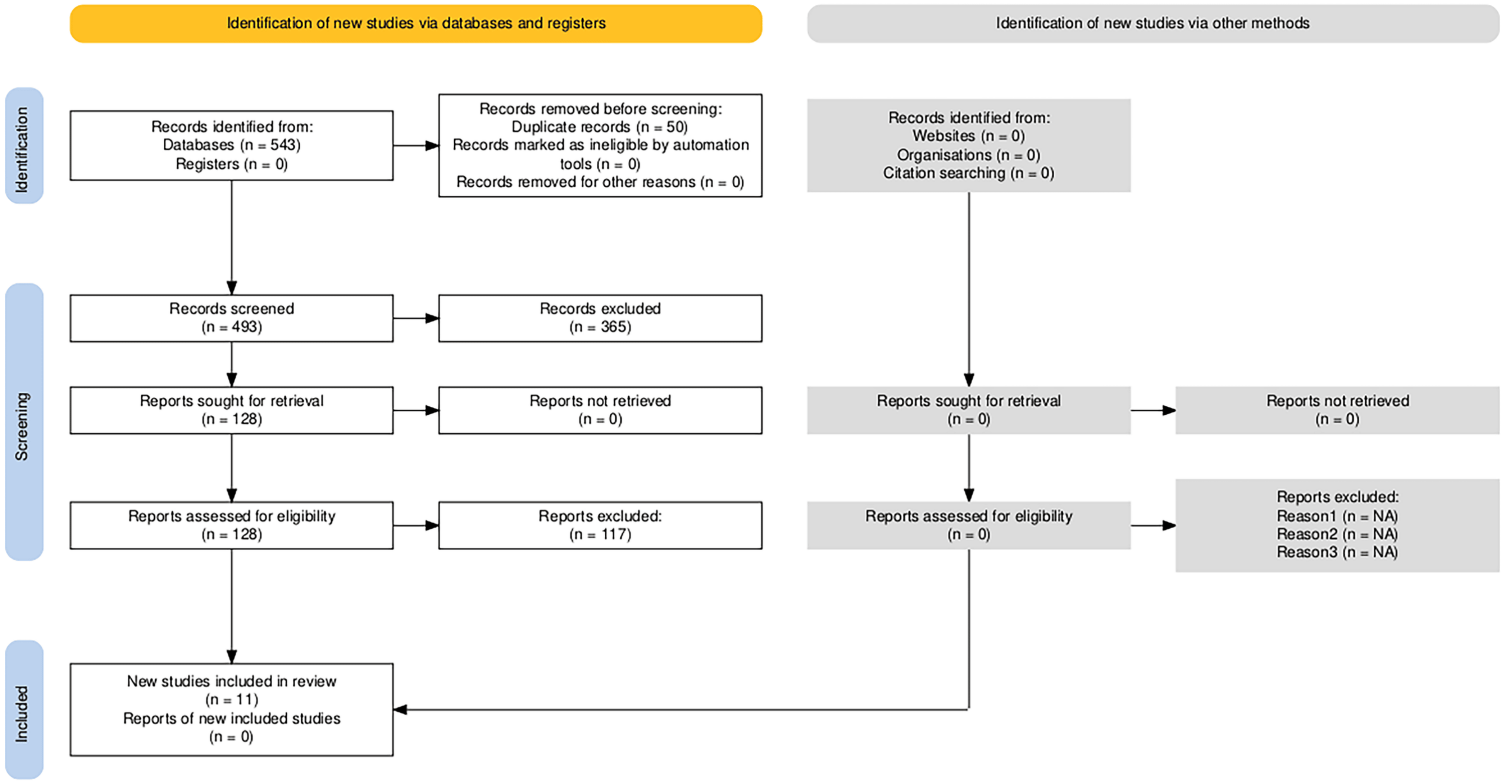
The PRISMA figures showing the steps to choose the studies for systematic review PRISMA: Preferred Reporting Items for Systematic Reviews and Meta-Analyses

This systematic review was conducted with over 543 studies in the literature review; however, only 11 studies were eligible for our inclusion criteria and included in this systematic review (Figure 1, Table 2). The studies included in this review employ a variety of research designs to explore the relationship between gut microbiota and biological therapies. Most studies utilize longitudinal and prospective cohort designs, with sample sizes ranging from small groups to larger cohorts, and focus on diverse therapeutic agents, including anti-TNF therapies, vedolizumab, and ustekinumab. Methodologies include 16S rRNA sequencing, quantitative polymerase chain reaction (qPCR) analysis, and in silico simulations to assess microbiota composition and its correlation with treatment efficacy and side effects. These studies contribute to a comprehensive understanding of how gut microbiota influences the therapeutic response and adverse events associated with biological treatments, providing valuable insights for personalized medicine approaches (Table 3).

**Table 2:** The different records (articles) retrieved using each database

Database/tool	Number of records retrieved
PubMed	122
Embase	98
Cochrane Library	52
Scopus	128
Google Scholar	117
ClinicalTrials.gov	26
Total	543

**Table 3:** Study characteristics and design overview CD: Crohn's Disease; UC: ulcerative colitis; IBD: inflammatory bowel disease; TNF: tumor necrosis factor; qPCR: quantitative polymerase chain reaction

Study	Design	Sample size	Therapies evaluated	Duration of follow-up	Analysis methodology
Zhou et al., 2018 [23]	Prospective cohort	72 CD, 51 UC, 73 healthy controls	Infliximab	30 weeks	16S rRNA gene sequencing, meta-analysis (PRISM, RISK cohorts)
Aden et al., 2019 [24]	Longitudinal, 2-step prospective cohort	12 IBD, 17 rheumatic diseases, 19 controls	Anti-TNF (infliximab), vedolizumab	2, 6, and 30 weeks	V3-V4 16S rRNA sequencing, metabolomic analysis
Busquets et al. 2021 [25]	Proof of concept, prospective cohort	38 IBD patients	Adalimumab, golimumab, infliximab	Baseline sampling	qPCR analysis of microbial markers
Höyhtyä et al., 2023 [26]	Longitudinal cohort study	10 responders, 19 non-responders (total 29)	Infliximab	Six weeks	16S rRNA sequencing, absolute microbial abundance assessment
Ananthakrishnan et al., 2017 [27]	Prospective study	85 IBD patients (43 UC, 42 CD)	Vedolizumab	14, 30, and 54 weeks	Metagenomic sequencing, neural network algorithm (violet) for predictive modeling
Effenberger et al., 2021 [28]	Longitudinal, prospective cohort study	65 IBD patients	Azathioprine, anti-TNF	12 and 30 weeks	16S rRNA sequencing, in-silico simulations
Kolho et al., 2015 [29]	Prospective study	68 pediatric IBD, 26 controls	Anti-TNF-α	Six weeks	Phylogenetic microarray, qPCR analysis
Shaw et al., 2016 [30]	Prospective cohort study	19 treatment-naïve pediatric IBD, 10 healthy controls	Various therapies	Variable	Generalized estimating equations, random forest classification
Rob et al., 2022 [31]	Longitudinal, observational study	11 IBD patients, 39 healthy controls	Ustekinumab	40 weeks	16S rRNA sequencing, biomarker analysis
Bazin et al., 2018 [32]	Longitudinal cohort study	19 spondylarthritis patients	Anti-TNF-α	Six months	qPCR analysis, microbial community profiling
Yeh et al., 2019 [33]	Prospective cohort, comparative study	34 psoriasis patients, 12 healthy controls	Secukinumab, ustekinumab	24 weeks	16S rRNA sequencing, comparative analysis of microbiota shifts

*Role of Gut Microbiota Composition on Biological Therapy Efficacy and Prediction of Its Respons*e

The studies reviewed reveal a nuanced relationship between gut microbiota composition and the efficacy of various biological therapies for autoimmune diseases. Research indicates that alterations in gut microbiota, such as increased abundance of specific bacterial taxa or shifts in microbial diversity, can significantly impact therapeutic outcomes. For instance, an increased abundance of Clostridiales has been associated with a better response to infliximab, while higher levels of Bifidobacterial have been linked to a more favorable response in pediatric IBD patients treated with infliximab. Similarly, the presence of certain bacterial species, such as *Roseburia inulinivorans*, has been identified as a marker for improved response to vedolizumab. These findings suggest that a tailored approach considering individual microbiota profiles may enhance therapeutic efficacy and personalized treatment strategies (Table 4). Regarding the prediction of biological treatment response according to gut microbiota composition, key microbial markers and community structures have been shown to correlate with the likelihood of achieving a clinical response. For example, specific bacterial groups, such as Clostridiales in infliximab-treated patients and butyrate-producing species in vedolizumab recipients, have demonstrated strong associations with treatment success. Advanced predictive models, including neural networks and multi-bacterial marker signatures, have achieved high accuracy in forecasting therapeutic outcomes. These predictive capabilities underscore the potential for integrating microbiota profiling into clinical practice to guide therapy selection and improve patient outcomes (Table 5).

**Table 4:** Studies on gut microbiota and efficacy of biologic therapies CD: Crohn's Disease; UC: ulcerative colitis; IBD: inflammatory bowel disease; TNF: tumor necrosis factor

Study	Therapy	Population	Gut microbiota alteration	Efficacy findings
Zhou et al., 2018 [23]	Infliximab	72 CD, 51 UC, 73 healthy controls (China)	Increased Actinobacteria and Proteobacteria, decreased Firmicutes	Clostridiales increase associated with infliximab response (93.8% accuracy).
Aden et al., 2019 [24]	Anti-TNF (infliximab, vedolizumab)	12 IBD, 17 rheumatic diseases, 19 controls (discovery); 23 IBD (validation)	The shift in microbiota toward healthy controls predicted butyrate synthesis linked to clinical remission	Metabolite network reconstruction suggested butyrate as a marker of remission post-anti-TNF therapy.
Höyhtyä et al., 2023 [26]	Infliximab	Pediatric IBD patients in Finland	Higher baseline abundance of Bifidobacterial in responders	Rapid response in patients with Bifidobacterial dominance.
Ananthakrishnan et al., 2017 [27]	Vedolizumab	43 UC, 42 CD patients	*Roseburia inulinivorans* and Burkholderiales species are higher in responders.	High baseline diversity linked to week 14 remission; don't neural networks use microbiome and clinical data to predict remission?
Effenberger et al., 2021 [28]	Azathioprine, anti-TNF	65 IBD patients	Decrease of Proteobacteria, increase of Bacteroidetes, Lactobacilli associated with persistent disease	In-silico simulation identified butyrate synthesis as predictive of therapeutic efficacy in IBD.
Kolho et al., 2015 [29]	Anti-TNF-α	68 pediatric IBD, 26 controls	Reduced microbial richness, abundance of butyrate producers, relative abundance of Gram-positive bacteria	Increased microbial diversity and similarity to controls in responders; specific bacterial groups predicted response to anti-TNF-α.
Shaw et al., 2016 [30]	Various therapies	19 treatment-naïve pediatric IBD, 10 healthy controls	Increased dysbiosis, specific genus-level differences between cases/controls and responders/non-responders	Pretreatment microbiome signatures predicted response with 76.5% accuracy.
Rob et al., 2022 [31]	Ustekinumab	11 IBD patients, 39 healthy controls	Differences in stool abundance of uncultured Subdoligranulum, Faecalibacterium, and Bacteroides	No significant change in biomarkers or microbiota composition in response to ustekinumab.
Bazin et al., 2018 [32]	Anti-TNF-α	19 spondylarthritis patients	No specific taxon modified; a higher proportion of Burkholderiales in responders	The taxonomic node before treatment predicted the clinical response; there was stable microbiota in the responders.
Yeh et al., 2019 [33]	Secukinumab, ustekinumab	34 psoriasis patients, 12 healthy controls	Secukinumab caused more profound alterations, including increased Proteobacteria and decreased Bacteroidetes.	Secukinumab therapy is associated with distinct gut microbiome shifts compared to ustekinumab.

**Table 5:** Studies on gut microbiota as predictors of treatment response CD: Crohn's Disease; UC: ulcerative colitis; IBD: inflammatory bowel disease; TNF: tumor necrosis factor

Study	Therapy	Population	Microbial predictors	Response predictive power
Zhou et al., 2018 [23]	Infliximab	16 CD patients (Subset of 72 CD, 51 UC)	Clostridiales abundance combined with calprotectin and CDAI levels	Prediction of infliximab response with 93.8% accuracy.
Busquets et al. 2021 [25]	Adalimumab, golimumab, infliximab	38 IBD patients	A 4-bacterial marker signature (qPCR analysis)	High predictive sensitivity (93.33%) and specificity (100%) for anti-TNF treatment response.
Höyhtyä et al., 2023 [26]	Infliximab	Pediatric IBD patients	Increased Bifidobacteriales, decreased Actinomycetales	Rapid response associated with Bifidobacteriales at baseline.
Ananthakrishnan et al., 2017 [27]	Vedolizumab	43 UC, 42 CD patients	*Roseburia inulinivorans* and Burkholderiales species; baseline microbiota diversity	The neural network algorithm (vedoNet), which combines microbiome and clinical data, has the highest accuracy in predicting treatment response.
Kolho et al., 2015 [29]	Anti-TNF-α	68 pediatric IBD, 26 controls	Specific bacterial groups predicting calprotectin levels	Bacterial groups predicted response to anti-TNF-α with an AUC of 0.85.
Shaw et al., 2016 [30]	Various therapies	19 treatment-naïve pediatric IBD	Specific genus-level differences; pretreatment microbiome signatures	76.5% accuracy for prediction of responder status.

The Influence of Gut Microbiota on Biological Therapy Side Effects

Research into the impact of gut microbiota on the side effects of biological therapies reveals that microbial alterations can influence adverse event profiles. For instance, shifts in microbiota related to metabolic pathways have been linked to therapeutic side effects. Specific studies indicate that dysbiosis and changes in bacterial metabolite production may contribute to the development of adverse events during treatment. Understanding these microbiota-related changes can help identify patients at risk for side effects and potentially guide adjustments in therapy to mitigate adverse outcomes (Table 6).

**Table 6:** Studies on gut microbiota and side effects/adverse events of biologic therapies

Study	Therapy	Population	Microbiota-related adverse events	Key findings on adverse events
Aden et al., 2019 [24]	Anti-TNF, vedolizumab	12 IBD, 17 rheumatic diseases, 19 controls; 23 IBD (validation)	Gut microbiota alteration potentially linked to metabolite dysregulation	Reduced butyrate synthesis and impaired metabolite networks are associated with poor response and potential side effects.
Ananthakrishnan et al., 2017 [27]	Vedolizumab	43 UC, 42 CD patients	Microbial function enrichment (e.g., amino acid synthesis) linked to therapy	Certain pathways, such as branched-chain amino acid synthesis, were enriched in patients, which may correlate with side effects or therapy resistance.

Discussion

This systematic review offers an in-depth examination of the role of gut microbiota in modulating the efficacy and side effect profile of biologic therapies for autoimmune diseases. The synthesis of current research highlights significant interactions between the gut microbiome and therapeutic outcomes, which underscores the potential for microbiota-based interventions in optimizing biological treatments, which is similar to what was reported in the literature [34-36].

The evidence indicates that gut microbiota composition can substantially influence the efficacy of biological therapies. Studies reveal that specific bacterial taxa and microbial diversity are closely linked to therapeutic responses. For instance, Zhou et al. (2018) demonstrated that an increased abundance of Clostridiales is associated with a positive response to infliximab in Crohn's disease patients. This finding suggests that microbiota profiles may serve as valuable biomarkers for predicting treatment success [23]. Similarly, Ananthakrishnan et al. (2018) identified higher levels of *Roseburia inulinivorans* and Burkholderiales species as markers for improved response to vedolizumab, emphasizing the role of specific microbial communities in therapeutic outcomes [27].

Further, research by Aden et al. (2019) highlights how metabolic predictions based on gut microbiota can inform treatment responses. Their study revealed that butyrate production, which is influenced by microbial community composition, correlates with clinical remission, suggesting that metabolic profiling of gut microbiota could guide therapeutic decisions [24]. This is corroborated by Höyhtyä et al. (2022), who found that pediatric IBD patients with a higher baseline abundance of Bifidobacteriales had a more rapid response to infliximab, suggesting that early microbial profiles can predict treatment success [26].

The review also underscores the role of gut microbiota in influencing the side effect profile of biological therapies. Studies have reported that alterations in the microbiota can impact the occurrence of adverse events. For instance, Aden et al. (2019) noted that reduced butyrate synthesis and impaired metabolite networks were associated with poor response and potential side effects during anti-TNF therapy [24]. Similarly, research by Ananthakrishnan et al. (2018) linked changes in microbial function, such as amino acid synthesis, with therapy-related side effects, suggesting that microbiota-mediated metabolic pathways might be involved in adverse events [27].

The predictive potential of gut microbiota for treatment response and adverse events is a notable finding from the reviewed studies. Busquets et al. (2020) identified a four-bacterial marker signature that effectively predicted treatment response to anti-TNF therapies with high sensitivity and specificity [25]. This is further supported by findings from Shaw et al. (2016), which demonstrated that pretreatment microbiome signatures could predict treatment outcomes, although not all studies found significant differences in dysbiosis between responders and non-responders [30]. These insights suggest that integrating microbiota profiles into clinical practice could enhance the personalization of biological therapies.

The collective evidence from these studies supports the hypothesis that modulating the gut microbiome can significantly impact both the efficacy and safety profile of biologic therapies. By understanding and leveraging the interactions between gut microbiota and treatment responses, clinicians can potentially optimize therapeutic strategies for autoimmune diseases. Future research should focus on validating these findings in larger cohorts and exploring the mechanisms through which microbiota influence drug metabolism and immune responses. Ultimately, a microbiome-based approach could lead to more personalized and effective management of autoimmune conditions, reducing adverse events and improving overall patient outcomes.

This systematic review is subject to several limitations. Firstly, the variability in study designs, methodologies, and patient populations across the included studies may impact the generalizability of the findings. Differences in microbiota profiling techniques, such as the use of 16S rRNA sequencing versus metagenomic approaches, can lead to inconsistencies in data interpretation. Additionally, the small sample sizes and short follow-up durations in some studies limit the robustness of the conclusions regarding long-term efficacy and safety. There is also a potential for publication bias, as studies with negative or inconclusive results may be underrepresented. Furthermore, while our review highlights associations between microbiota and treatment outcomes, causality cannot be firmly established due to the observational nature of most included studies. Future research with standardized methodologies and larger, more diverse cohorts is needed to address these limitations and provide more definitive insights.

## Conclusions

In conclusion, the evidence gathered from this systematic review underscores the critical role of gut microbiota in influencing the efficacy and side effect profile of biologic therapies for autoimmune diseases. The observed associations between specific microbial profiles and treatment outcomes suggest that microbiota modulation could be a promising strategy for enhancing therapeutic efficacy and reducing adverse events. Personalized approaches that integrate microbiota assessments into treatment planning may offer significant improvements in patient management and outcomes. Continued research is essential to validate these findings and explore the underlying mechanisms, paving the way for more effective and tailored interventions in autoimmune disease therapy.
